# Knowledge, attitudes and practices of clinicians in Shenzhen regarding pediatric streptococcal pharyngitis diagnosis and treatment

**DOI:** 10.3389/fcimb.2026.1712298

**Published:** 2026-03-02

**Authors:** Shuting Zhuang, Danchun Guo, Yifan Ruan, Li Li, Yanmin Bao, Wenjian Wang, Dingle Yu

**Affiliations:** Shenzhen Children’s Hospital, Shantou University Medical College, Shenzhen, China

**Keywords:** child, group A *streptococcus*, knowledge, attitudes and practices, pharyngitis, Shenzhen

## Abstract

**Background:**

The objective of this study was to investigate the current status of clinicians’ knowledge, attitudes and practices (KAP) regarding the diagnosis and management of pediatric group A *Streptococcus* (GAS) pharyngitis and the factors influencing them in Shenzhen, Guangdong Province, China.

**Methods:**

For this cross-sectional study, an online questionnaire (12 KAP items, 6 descriptive items) developed using the Delphi method was administered to 190 practicing clinicians in Shenzhen from February 23 to February 25, 2025. The data were analyzed using t tests and analysis of variance, with comparisons performed according to demographic characteristics and, after KAP dichotomization (≥ 75% threshold), hospital type. Pearson correlations between KAP scores were assessed, and significant univariate predictors of KAP were examined using multiple linear regression analysis.

**Results:**

Knowledge score correlated with practice score (r = 0.351, *p* < 0.05), but attitude score did not correlate with knowledge score (r = 0.048, *p* > 0.05) and practice score (r = 0.072, *p* > 0.05). Respondents’ knowledge was influenced by factors such as guideline exposure, the hospital level, and age (all *p* < 0.05). Their practices were influenced by factors such as guideline exposure, the hospital level, and years of practice (all *p* < 0.05). Systematic guideline exposure had a positive predictive effect on respondents’ knowledge levels (*p* < 0.05). The practice scores of individuals with more than 20 years of practice were significantly higher than those of individuals with 4–20 years of practice (*p* < 0.05).

**Conclusion:**

The present study showed that clinicians in Shenzhen have a relatively advanced level of expertise and positive attitudes regarding the diagnosis and management of GAS pharyngitis in children. However, the extent of their knowledge was influenced by their degree of systematic exposure to diagnostic and treatment guidelines, and their practice expertise was constrained by their experience, potentially exacerbating antibiotic resistance. Standardized guideline application should be strengthened and clinicians’ practical skills should be improved to enhance guideline adherence and promote high-quality care delivery.

## 1 Introduction

Group A *Streptococcus* (GAS), also known as *Streptococcus pyogenes*, is the primary pathogen responsible for acute bacterial pharyngitis in children. It causes non-invasive (e.g., GAS pharyngitis) and invasive disease, as well as nonsuppurative sequelae, such as acute rheumatic fever (ARF) (Gerber et al., 2009). ARF and its sequela rheumatic heart disease (RHD) persist as substantial public health challenges in developing and low-income countries (Liang et al., 2023; Rwebembera and Beaton, 2024; Watts et al., 2025). Globally, an estimated 288.6 million cases of GAS pharyngitis occur annually among children aged 5–14 years (Miller et al., 2022). In regions with historically high incidence rates and limited healthcare resources, the combined prevalence of ARF is approximately 18.4 cases per 1000 people (Abdu et al., 2024). The estimated annual incidence of RHD worldwide in 2021 was 3.85 million cases (Tang et al., 2025). Additionally, RHD carries a relatively high mortality rate, with the latest data indicating that approximately 7.5 out of every 1000 patients worldwide die each year (Lupieri et al., 2025). These data underscore the importance of early intervention to prevent GAS infection (Carapetis et al., 2005; Yu et al., 2023).

In recent years, the increased number of GAS infections in the United Kingdom and other European nations has caused global concern (Wrenn et al., 2023; Venkatesan, 2023; Pigeolet et al., 2023; Flores, 2025). In China, the focus on the clinical management of GAS pharyngitis has increased, evidenced by a marked increase in the number of relevant research publications in the Chinese Medical Association Publishing House. The number of articles published between 2022 and May 2025 was more than threefold that published between 2016 and 2021 (Chinese Medical Association Publishing House, 2025). However, the standardization of this disease’s diagnosis and treatment faces numerous challenges. The primary challenge is that GAS pharyngitis and viral pharyngitis have very similar clinical features and are difficult to distinguish reliably, but the strategies for their treatment are distinct (Shulman et al., 2012). Furthermore, the clinical diagnosis of GAS pharyngitis and scarlet fever in China is frequently accompanied by irregularities, particularly in cases involving atypical rash manifestations. In such cases, the lack of a standardized assessment process or pathogen test for scarlet fever can lead to an elevated risk of misdiagnosis or missed diagnosis (Yu et al., 2020; National Center for Pediatric Infectious and Allergic Disease Surveillance et al., 2025). In addition, clinicians’ inadequate awareness regarding antimicrobial selection principles (e.g., the high rate of resistance to macrolide antimicrobials in China) may have significant impacts on treatment outcomes and may even indirectly increase the risk of ARF (Yu et al., 2024, 2021; Chan et al., 2019). Moreover, the use of ancillary tests [e.g., rapid antigen detection tests (RADTs) and throat culture] and clinical scoring systems (e.g., the Centor and McIsaac scores) is not widespread in China’s primary healthcare system, which may lead to over- or under-treatment (National Center for Pediatric Infectious and Allergic Disease Surveillance et al., 2025; Miron et al., 2025).

Although clear guidelines for GAS pharyngitis diagnosis, antibiotic selection, and treatment duration have been established, international studies have revealed suboptimal adherence to them (Di Muzio et al., 2020; Alkhazi et al., 2018; Hedin et al., 2014). To date, no research on this topic has been conducted in China. In this case study, the knowledge, attitudes, and practices (KAP) of clinicians in Shenzhen, Guangdong Province, China, regarding the diagnosis and treatment of GAS pharyngitis in children, as well as potential influencing factors, were investigated. Ultimately, we aimed to provide a scientific basis for the improvement of adherence to GAS pharyngitis guidelines and promotion of the provision of high-quality diagnostic and treatment services.

## 2 Methods

### 2.1 Study setting

Shenzhen is one of the most rapidly developing cities and a hub for GAS research in China. Thus, the performance of this case study in this location has distinct advantages. Firstly, 15.1% of its residents are children (Shenzhen Government Online, 2021), and the population is characterized by significant mobility (Tian et al., 2022). Secondly, a disparity exists in the city’s tertiary healthcare system, characterized by the concentration of high-quality resources and strong demand at the primary care level (Tian et al., 2022). Thirdly, the region’s hot and humid climate contributes to a relatively high incidence of pharyngitis (Majeed et al., 1987).

### 2.2 Participants

An online questionnaire was distributed to physicians in Shenzhen engaged in clinical work from February 23 to February 25, 2025. The exclusion criteria were non-Shenzhen practitioner location, questionnaire completion time < 105 seconds (a threshold determined from pilot testing to indicate insufficient engagement), and incomplete responses.

Considering a potential 20% rate of invalid questionnaires, the required sample size was set to be 5 to 10 times the number of scale items. With 12 KAP items and 6 descriptive items, this resulted in 225 valid questionnaires (10 times).

### 2.3 Methodology

#### 2.3.1 Questionnaire design

**Initial scale development.** The questionnaire was designed based on the relevant international literature (Shulman et al., 2012; Di Muzio et al., 2020; Ashurst et al., 2025) and the KAP theoretical model (Kalaimani et al., 2023). Three dimensions containing a total of 23 items were initially defined. A two-round Delphi study was conducted with seven experts specializing in GAS pharyngitis with more than 10 years of clinical experience and master’s degrees or higher education levels. The questionnaire and its evaluation criteria were sent to the experts via email. They rated the relevance and importance of each item using a 5-point Likert scale (higher scores reflected greater relevance and importance). Space for qualitative feedback was also provided. In round 1, five items with scores < 3.50 and coefficients of variation (CVs) > 0.25 were eliminated. In round 2, performed to clarify ambiguous rationales for the inclusion of some retained items, all remaining items met the validity thresholds (scores > 3.50, CVs < 0.25). Consensus was confirmed by Kendall’s *W* concordance (relevance: *W =* 0.62, *p* < 0.001; importance: *W =* 0.60, *p* < 0.001). The expert authority was robust {judgment coefficient [Ca] = 0.91, familiarity coefficient [Cs] = 0.79, authority coefficient [(Ca + Cs)/2] = 0.85}, supported by high response rates [round 1: 88% (*n* = 7/8); round 2: 100% (*n* = 7/7)]. Ultimately, the initial 17-item scale included 1 descriptive item.

**Small-scale pilot survey**. Fifty questionnaires were distributed to clinicians in Shenzhen using randomized sampling. 45 complete responses were analyzed. Five issues affecting reliability were identified. The final questionnaire consisted of 12 KAP items and 6 descriptive items. During the pilot phase, the internal consistency was acceptable for attitude (Cronbach’s *α* = 0.62) and knowledge (Cronbach’s *α* = 0.77).

#### 2.3.2 Questionnaire content

The first section of the questionnaire consists of six items that solicit demographic information, including respondents’ sex, age, education level, years of work experience, employer name, and hospital level. The second part contains 12 items in three dimensions: knowledge, attitudes, and practices. The knowledge dimension contains four true (2 points)/false (1 point) questions. The attitude dimension contains four questions, with responses structured by a 5-point Likert-scale ranging from “strongly disagree” (1 point) to “strongly agree” (5 points). The practice dimension contains one multiple-choice question (1 or 2 points), one “yes/would” (2 points)/”no/would not” (1 point) question, and two single-choice questions (correct, 2 points; incorrect, 1 point). Dimension scores were calculated using the formula: (actual score/maximum possible score) × 100%.

#### 2.3.3 Survey method

An invitation to participate in the survey was distributed to WeChat groups of physicians in Shenzhen via the Wenjuanxing platform (https://www.wjx.cn). The questionnaire was accessed via a WeChat QR code or mini-program.

Clinical trial number: not applicable.

#### 2.3.4 Statistical analysis

Data processing and analysis were performed using WPS Office (Kingsoft Office, Beijing, China) and SPSS Statistics (version 22.0; IBM, Armonk, NY, USA). Continuous data are presented as means ± standard deviations. Categorical data are expressed as frequencies and percentages. Group comparisons based on demographic characteristics (e.g., education level, years of work experience, and hospital level) were performed using independent-samples *t* test (for two groups) and analysis of variance (ANOVA, for three or more groups). Additionally, following dichotomization using a ≥ 75.0% score threshold, the independent-samples *t* test was used to examine differences in KAP scores according to hospital type. Bivariate linear relationships between KAP scores were analyzed using Pearson’s correlation coefficient (*r*). Subsequently, potential predictors (exposure to guidelines, the medical institution level and years of experience) were screened via univariate analysis (ANOVA and the independent-samples *t* test). A multiple linear regression analysis was then performed to identify factors associated independently with KAP scores. Statistical significance was defined as *p* < 0.05.

### 2.4 Retrospective prescription pattern analysis

To complement the survey findings with objective prescribing data, a retrospective analysis was performed on the electronic medical records of Shenzhen Children’s Hospital. This analysis evaluated antibiotic prescription patterns for outpatient children with positive RADTs in December 2021 and December 2024.

## 3 Results

### 3.1 Participant characteristics

In total, 228 questionnaires, including those from the pre-survey phase, were collected. After the exclusion of 38 questionnaires according to the defined criteria (1 for non-Shenzhen location, 1 for incompleteness, and 36 for completion time < 105 seconds), 190 valid questionnaires remained (83.3% response rate). Of the 190 physicians practicing in Shenzhen who participated in the survey, 65.3% (*n* = 124) were female, 80% (*n* = 152) were aged 30–50 years, 59.5% (*n* = 113) held master’s degrees or higher qualifications, 56.3% (*n* = 107) had more than 10 years of professional experience, and 79.5% (*n* = 151) worked at tertiary hospitals (**Table 1**).

**Table 1:** Demographic characteristics of survey participants (*n* = 190).

Characteristic		*n*	%
Sex	Male	66	34.7%
	Female	124	65.3%
Age	< 30 years	20	10.5%
	30–50 years	152	80.0%
	> 50 years	18	9.5%
Highest degree	Bachelor’s	77	40.5%
	Master’s	102	53.7%
	Doctorate	11	5.8%
Years of work experience	1–3	26	13.7%
	4–5	15	7.9%
	6–10	42	22.1%
	11–20	64	33.7%
	> 20	43	22.6%
Hospital classification	Class I	38	20.0%
	Class II	1	0.5%
	Class III grade A	151	79.5%

### 3.2 KAP

Mean KAP scores were 7.48 ± 1.09 (93.5%), 16.34 ± 2.98 (81.7%), and 6.46 ± 0.86 (80.8%), respectively (**Table 2**). In the knowledge dimension, 8.4% of respondents lacked an understanding of the main symptoms and signs of pediatric GAS pharyngitis, 14.7% lacked an understanding of diagnostic studies, and 8.4% lacked an understanding of antibiotic selection. 20% of respondents were unfamiliar with the appropriate evidence-based duration of antibacterial therapy. In the attitude dimension, only 33.2% of the respondents strongly agreed with the use of scoring systems when evaluating children with suspected bacterial pharyngitis, and 38.9% strongly agreed with the performance of throat swab culture when rapid antigen test results were negative. In the practices dimension, 53.2% of respondents favored testing for antibodies such as antistreptolysin O (ASO) as an adjunctive tool for the diagnosis of pediatric GAS pharyngitis. 20 respondents (6.3%) preferred macrolides (e.g., erythromycin and azithromycin) as first-line antimicrobial therapy. Two-thirds of these clinicians cited the lower risk of antibiotic resistance as their primary rationale for this preference.

**Table 2:** KAP scores.

Question	Score (mean ± SD)
Knowledge dimension^a^	
Are you familiar with the key symptoms and signs of pediatric GAS pharyngitis?	1.92 ± 0.28
Are you familiar with common diagnostic studies of pediatric GAS pharyngitis?	1.85 ± 0.36
Are you familiar with guideline-concordant antibiotic selection for the treatment of pediatric GAS pharyngitis?	1.92 ± 0.28
Are you familiar with the evidence-based duration of antibacterial therapy for pediatric GAS pharyngitis?	1.80 ± 0.40
Total score	7.48 ± 1.09
Attitude dimension^b^	
When you have a child with suspected bacterial pharyngitis, do you apply scoring systems?	3.61 ± 1.26
When you have a child with suspected bacterial pharyngitis, do you perform a rapid test for GAS?	4.23 ± 1.13
When you have a child with suspected bacterial pharyngitis, if the GAS rapid antigen test is negative, do you perform a throat swab culture?	3.68 ± 1.31
Do you consider evidence-based antimicrobial therapy essential for the management of pediatric GAS pharyngitis?	4.82 ± 0.53
Total score	16.34 ± 2.98
Practices dimension^c^	
Which diagnostic studies would you perform to confirm pediatric GAS pharyngitis?	1.39 ± 0.49
Which antibiotic do you use to treat pediatric GAS pharyngitis?	1.86 ± 0.35
For how many days do you prescribe antibiotic therapy for the treatment of pediatric GAS pharyngitis?	1.65 ± 0.48
Have you ever encountered cases of ARF or acute post-streptococcal glomerulonephritis resulting from the suboptimal management of pharyngitis in your clinical practice?	1.56 ± 0.50
Total score	6.46 ± 0.86

a Maximum item score = 2 points, maximum total score = 8.

b Maximum item score = 5 points, maximum total score = 20.

c Maximum item score = 2 points, maximum total score = 8.

### 3.3 KAP determinants

Exposure to guidelines or expert consensus statements on GAS pharyngitis was a significant determinant of the knowledge and practice scores (both *p* < 0.05). The medical institution level and years of experience were also main factors affecting these scores (both *p* < 0.05).

Exposure to guidelines and expert consensus statements had a positive predictive effect on knowledge scores (*B* = 1.13, *β* = 0.49, *p* < 0.001), with this factor explaining 25.6% of the model variance. The practice scores of clinicians with more than 20 years of professional experience were significantly higher than those of clinicians with 4–5 years (*B* = 0.52, *β* = 0.16, *p* < 0.05), 6–10 years (*B* = 0.38, *β* = 0.18, *p* < 0.05), and 11–20 years (*B* = 0.38, *β* = 0.21, *p* < 0.05) of experience. This factor accounted for 6.7% of the model variance (**Table 3**–**5**).

**Table 3:** Factors associated with knowledge scores.

Predictor	*n*	Score (mean ± SD)	Univariate analysis results		Multivariable linear regression results		
			*F/t*	*p*	*β*	95% Cl	*p*
Sex			–0.62	0.425			
Male	66	7.41 ± 1.11					
Female	124	7.52 ± 1.08					
Age group, years			3.12	0.047			
0–29	20	7.80 ± 0.70			0.00		
30–50	152	7.39 ± 1.17			0.09	–0.83, 0.06	0.086
>50	18	7.94 ± 0.24			0.55	–0.79, 0.42	0.547
Highest degree			0.07	0.931			
Bachelor’s	77	7.49 ± 1.06					
Master’s	102	7.49 ± 1.08					
Doctorate	11	7.36 ± 1.43					
Years of work experience			1.57	0.183			
1–3	26	7.73 ± 0.72					
4–5	15	7.27 ± 1.44					
6–10	42	7.38 ± 1.13					
11–20	64	7.31 ± 1.30					
> 20	43	7.77 ± 0.61					
Hospital classification			4.41	0.014			
Class I	38	7.13 ± 1.30			–0.06	–0.50, 0.19	0.363
Class II	1				0.01	–1.68, 2.04	0.851
Class III grade A	151	7.57 ± 1.02			0		
Exposure to relevant guidelines/expert consensus			67.60	< 0.001			
No	62	6.69 ± 1.41			0		
Yes	128	7.87 ± 0.46			0.49	0.83, 1.42	< 0.001

**Table 4:** Factors associated with attitude scores.

Predictor	*n*	Score (mean ± SD)	Univariate analysis results	
			*F/t*	*p*
Sex			–0.90	0.221
Male	66	16.09 ± 3.30		
Female	124	16.48 ± 2.79		
Age group, years			0.24	0.783
0–29	20	16.50 ± 2.93		
30–50	152	16.38 ± 3.01		
>50	18	15.89 ± 2.89		
Highest degree			0.69	0.502
Bachelor’s	77	16.04 ± 3.17		
Master’s	102	16.53 ± 2.84		
Doctorate	11	16.73 ± 2.83		
Years of work experience			0.55	0.700
1–3	26	16.15 ± 3.54		
4–5	15	16.07 ± 3.47		
6–10	42	16.62 ± 2.45		
11–20	64	16.63 ± 2.78		
> 20	43	15.86 ± 3.23		
Hospital classification			0.23	0.797
Class I	38	15.76 ± 3.38		
Class II	1			
Class III grade A	151	16.46 ± 2.85		
Exposure to relevant guidelines/expert consensus			1.01	0.316
No	62	16.00 ± 3.14		
Yes	128	16.51 ± 2.89		

**Table 5:** Factors associated with practice scores.

Predictor	n	Score (mean ± SD)	Univariate analysis results		Multivariable linear regression results		
			F/t	p	b	95% Cl	p
Sex			-0.66	0.739			
Male	66	6.41 ± 0.88					
Female	124	6.49 ± 0.86					
Age group, years			1.09	0.338			
0-29	20	6.55 ± 0.69					
30-50	152	6.42 ± 0.90					
51-90	18	6.72 ± 0.75					
Highest degree			0.49	0.611			
Bachelor’s	77	6.39 ± 0.79					
Master’s	102	6.52 ± 0.92					
Doctorate	11	6.45 ± 0.82					
Years of work experience			2.74	0.030			
1-3	26	6.69 ± 0.74			-0.02	-0.46, 0.37	0.829
4-5	15	6.20 ± 1.01			-0.16	-1.02, -0.03	0.038
6-10	42	6.33 ± 0.98			-0.18	-0.74, -0.01	0.042
11-20	64	6.33 ± 0.82			-0.21	-0.70, -0.05	0.025
> 20	43	6.74 ± 0.76			0.000		
Hospital classification			3.21	0.043			
Class I	38	6.21 ± 0.70			-0.13	-0.59, 0.03	0.073
Class II	1				-0.12	-3.14, 0.19	0.081
Class III grade-A	151	6.54 ± 0.89			0		
Exposure to relevant guidelines/expert consensus			4.17	0.043			
No	62	6.27 ± 0.89			0		
Yes	128	6.55 ± 0.84			0.11	-0.06, 0.47	0.120

### 3.4 Interrelationships and institutional influences on KAP scores

A significant positive correlation between the knowledge and practice scores was identified (r = 0.351, *p* < 0.05). No significant correlation was found between the attitude and knowledge scores (r = 0.048, *p* > 0.05), nor between the attitude and practice scores (r = 0.072, *p* > 0.05; **Table 6**).

**Table 6:** KAP score correlations.

	Knowledge score	Attitude score	Practice score
Knowledge score	1.00	0.048	0.351**
Attitude score	0.048	1.00	0.072
Practice score	0.351**	0.072	1.00

**Two-tailed p < 0.01.

Significant disparities in attitude and practice scores were observed between respondents working at the pediatric specialty hospital in Shenzhen and those working at other tertiary hospitals (**Table 7**); the scores of the former group were higher (attitude: *t* = –1.17, *p* = 0.02; practice: *t* = –1.46, *p* = 0.003).

**Table 7:** Associations of KAP scores with hospital type.

Score	Shenzhen Children’s Hospital (*n* = 73)	Other tertiary hospitals (*n* = 53)	*t*	*p*
Knowledge	1.93 ± 0.25	1.89 ± 0.32	0.87	0.082
Attitude	1.81 ± 0.40	1.72 ± 0.46	1.17	0.020
Practice	1.89 ± 0.36	1.79 ± 0.41	1.46	0.003

### 3.5 Determinants of antimicrobial agent selection

The factors reported by respondents to influence their antimicrobial selection for the treatment of pediatric GAS pharyngitis were: a preference for empirical antibiotic therapy (81.1%), clinical efficacy (82.6%), the low propensity for resistance development (61.6%), the minimized dosing frequency or short treatment duration (52.1%), hospital formulary restrictions on antimicrobial agents (50.0%), the lower financial burden (48.4%), insurance reimbursement policies (46.3%), the lack of a skin test mandate (36.8%), and parental demand for specific antibiotics (9.5%; **Table 8**). More than half (58.3%) of the respondents who expressed a preference for macrolide antibiotics cited the low propensity for resistance development as an influencing factor.

**Table 8:** Attitudes toward GAS vaccination.

Response	*n*	Percentage
Strongly agree	105	55.3%
Agree	46	24.2%
Neutral	35	18.4%
Disagree	3	1.6%
Strongly disagree	1	0.5%

### 3.6 Observed antibiotic prescription patterns

The retrospective analysis included 90 cases in December 2021 and 91 cases in December 2024, with positive RADTs and diagnoses of pharyngitis. Cephalosporins were the most commonly prescribed antibiotics, accounting for 63.0% of the prescriptions. Within this class, the first-generation antibiotic cefadroxil accounted for 37.6%. Penicillin-class agents constituted 32.0% of prescriptions, predominantly amoxicillin-clavulanate (30.0%), with amoxicillin accounting for 2.0%. The remaining 5.0% comprised macrolides and clindamycin.

### 3.7 Clinicians’ perspectives and knowledge application

The majority (55.3%) of the surveyed clinicians strongly agreed that GAS vaccination conferred significant benefits in the prevention and control of GAS pharyngitis. Only 0.5% disagreed with this statement (**Table 8**). The positive attitude toward GAS vaccination aligned with the majority (67.4%) of respondents’ engagement with guidelines and expert consensus statements. Only 2.6% of clinicians selected an incorrect definition of GAS pharyngeal carriage. The respondents demonstrated a marked preference for tonsil preservation over tonsillectomy in cases of pediatric GAS pharyngitis (**Table 9**).

**Table 9:** Association between pediatric GAS pharyngitis recurrence frequency and tonsillectomy.

Annual pediatric GAS pharyngitis recurrence episodes (n)	Tonsillectomy recommendations (n)	Tonsillectomy non-recommendations (n)
1–3	18	73
4–6	7	19
7–10	2	4
> 10	2	7
Total	29	103

## 4 Discussion

Acute pharyngitis is the most common infectious disease in children. GAS causes approximately 20–30% of pediatric pharyngitis cases. The incidence of GAS pharyngitis peaks during school age, especially in children aged 7–8 years (Centers for Disease Control and Prevention, 2022). Typically, the disease presents acutely with high fever (temperature ≥ 39°C), the sudden onset of sore throat and swallowing pain, and systemic symptoms such as headache, abdominal pain, myalgia, nausea, vomiting, and malaise. Physical examination reveals erythema of the posterior pharyngeal wall, congested and patchy tonsillopharyngeal exudates (which are easily wiped away), palatal petechiae, tender nodes of anterior cervical adenitis, and scarlatiniform rash (Shulman et al., 2012). However, the classic presentation of GAS pharyngitis is uncommon in children aged < 3 years (Chiappini et al., 2012). The diagnosis of this disease requires a detailed history, meticulous physical examination, and initial screening using clinical prediction tools (such as the Centor and McIsaac scores). Definitive diagnosis relies on etiological confirmation through methods including RADTs, throat culture, and nucleic acid amplification techniques. A 10-day course of penicillins is the first-line treatment to prevent ARF and RHD. In the case of penicillin allergy, cephalosporins are considered an alternative option (Hirani et al., 2025; Pellegrino et al., 2023; Chiappini et al., 2024; National Center for Pediatric Infectious and Allergic Disease Surveillance et al., 2025). The guidelines of the World Health Organization and numerous national bodies for the management of acute pharyngitis explicitly classify GAS pharyngitis as a condition requiring standardized antibiotic therapy (World Health Organization, 2022).

The study participants had high levels of knowledge about the management of pediatric GAS pharyngitis and robust confidence in vaccine-based prophylactic strategies. This is due primarily to recent nationwide efforts to disseminate relevant guidelines and expert consensus statements (National Center for Pediatric Infectious and Allergic Disease Surveillance et al., 2025; Yu et al., 2022), and may also be related to Shenzhen’s status as a hub for GAS research in China, which potentially enhances local physicians’ timely access to guidelines.

Further improvement in the efficiency of translating attitudes into practice is needed. In this study, scores for these dimensions did not correlate. The translation of attitude into practice may be significantly hampered by external constraints. These barriers manifest most prominently as over-reliance on clinical experience during decision making (Croskerry, 2020), significant patient heterogeneity, and disparities in healthcare resource allocation (Norton et al., 2018). For instance, more than half of the clinicians participating in this study who preferred macrolides as the first-line treatment for GAS pharyngitis perceived that these drugs carried a lower risk of resistance development, contradicting data on resistance in China (Chan et al., 2019; Zhang et al., 2019). This discrepancy suggests that empirical prescription persists despite clinicians’ acceptance of guideline recommendations, likely due to diagnostic accessibility limitations. Additionally, variations in parents’ understanding of treatment duration complicate adherence (Norton et al., 2018). To address this gap, standardized clinical supervision is recommended to improve guideline adherence (Croskerry, 2020). Concurrently, the establishment of an “e-prescribing platform with alert functionality” based on real-time regional resistance data (Luo and Gellad, 2023; Zhou et al., 2023) and the strengthening of patient education initiatives targeting caregivers’ knowledge of GAS pharyngitis (Ralph et al., 2022; Yadav et al., 2024) have been proposed.

Regarding diagnostic testing practices, only 33.2% of the clinicians who participated in this study strongly agreed with the use of scoring systems when evaluating children with suspected bacterial pharyngitis. This result stands in stark contrast to the widespread adoption of this practice in high-income countries. Scoring tools have been recognized to enhance the diagnostic sensitivity for GAS pharyngitis while reducing antimicrobial prescription rates and the burden of RADTs, thereby reducing healthcare costs (ESCMID Sore Throat Guideline Group et al., 2012; Miron et al., 2025). This discrepancy is likely attributable to the absence of a universally applicable validated clinical prediction rule for GAS infection diagnosis in China, combined with insufficient clinical awareness. Consequently, there is an urgent need to embed clinical scores in diagnostic pathways. In addition, only 38.9% of physicians strongly agreed with the performance of confirmatory throat swab culture following negative RADT results for children with suspected bacterial pharyngitis, indicating the misalignment of practice with guideline recommendations. International guidelines differ, with European guidelines advising against routine culture confirmation after a negative RADT result (Chiappini et al., 2012), United States guidelines recommend such confirmation for children and adolescents (Shulman et al., 2012). China tends to favor the latter approach (National Center for Pediatric Infectious and Allergic Disease Surveillance et al., 2025), but the study result demonstrates inadequate clinical adherence to this recommendation. Moreover, 53.2% of the study respondents preferred testing for antibodies such as ASO for the diagnosis of pediatric GAS pharyngitis. However, guidelines generally discourage the routine use of ASO testing for the diagnosis of acute pharyngitis, as this antibody typically becomes detectable in plasma 2–4 weeks after GAS infection (Ashurst et al., 2025; Pavez et al., 2019), and an elevated ASO titer does not necessarily indicate the presence of an acute infection requiring antimicrobial therapy (Gewitz et al., 2015; Parks et al., 2015). Thus, the dissemination of expert consensus guidelines emphasizing the standardized application of clinical prediction tools and pathogen detection is important (Ashurst et al., 2025). The development and implementation of intelligent clinical decision support templates in electronic health records systems is also recommended. These tools should automatically flag the indication for confirmatory throat swab culture for children with negative RADT results (Alowais et al., 2023). Furthermore, discussion of the applicability and diagnostic deficiencies of ASO antibody testing should be incorporated into the mandatory physician training programs.

Clinical efficacy and a preference for empirical antibiotic therapy were fundamental criteria underlying respondents’ clinical decision making about antimicrobial agents. The study respondents prioritized clinical efficacy in their decision making (82.6%). In this context, several key points must be emphasized. First, as antimicrobial agents are ineffective against viral pharyngitis (Ashurst et al., 2025), treatment efficacy depends fundamentally on accurate pathogen (e.g., GAS) identification through diagnostic tests such as RADTs (Kramme et al., 2024). Second, symptom relief does not equate with microbial eradication. The completion of the full antimicrobial therapy course is essential for GAS clearance, preventing recurrence and serious complications such as ARF (Shulman et al., 2012; Ashurst et al., 2025; Spinks et al., 2021).

The second major factor influencing the study respondents’ clinical decision making was a preference for empirical antibiotic therapy (81.1%). The empirical treatment of GAS pharyngitis is indeed necessary and prevalent in primary care settings and resource-limited environments, where access to confirmatory tests such as RADTs may be lacking. However, the over-reliance on empirical therapy, especially in the absence of confirmed pathogen identification, leads to unnecessary antimicrobial exposure and thus exacerbates the development of bacterial antimicrobial resistance (Teratani et al., 2019).

Our retrospective outpatient data demonstrate a prescribing preference. Cephalosporins were prescribed in 63.0% of GAS-positive cases, compared with 32.0% for agents requiring penicillin skin testing (PST). This preference aligns with the finding in our survey that approximately 80% of physicians who chose cephalosporins as first-line treatment indicated that the convenience of skipping allergy testing was a factor influencing this choice. Chinese protocols mandate universal PST prior to the administration of the drug (National Health Commission of the People’s Republic of China, 2021), whereas Western guidelines explicitly discourage routine PST (World Health Organization, 2021), as a positive PST result does not diagnostically equate to penicillin allergy (Copaescu et al., 2023). This practice is intrinsically linked to China’s unique healthcare context and paradigms, characterized by defensive medicine practices driven by China’s medicolegal landscape (Jiang et al., 2023), the clinical accessibility of alternative antimicrobial agents (e.g., macrolides) (Jiang et al., 2022) and cephalosporin safety profiles (National Health Commission of the People’s Republic of China, 2021). Although recent initiatives advocate PST de-implementation (National Health Commission of the People’s Republic of China, 2021), the comprehensive integration of such policies requires sustained systemic reinforcement. Thus, clinicians need to maintain updated knowledge of evidence-based guidelines (Beaton et al., 2020), apply it to standardize clinical practices, and follow recommendations that prioritize microbiologically guided decision making and standardized penicillin allergy assessment for antimicrobial prescription (Jiang et al., 2022). In addition, healthcare services need to be strengthened to ensure that providers have adequate resources and skills to diagnose and appropriately manage GAS infections (Beaton et al., 2020).

Decisions about the performance of tonsillectomy in children should be made with careful consideration of the specific national context to optimize surgical indications. The relevant expert consensus statement for China explicitly states that tonsillectomy for the sole purpose of reducing the frequency of GAS pharyngitis recurrence is not recommended (National Center for Pediatric Infectious and Allergic Disease Surveillance et al., 2025). This stance contrasts with some historical practices, but aligns with the current global medical trend, which increasingly favors a conservative approach. Currently, the international medical community strongly emphasizes that tonsillectomy should generally be reserved for cases in which conservative treatment has proven to be ineffective or in which symptoms severely affect patients’ quality of life (Jones et al., 2024; O’Hara et al., 2024; Shaw et al., 2025). This call to exercise caution in some settings (potentially including China) may stem from perceived inadequacies of postoperative follow up. Thus, prospective cohort studies and the systematic tracking of post-tonsillectomy outcomes (i.e., recurrent infection rates, complication incidences, and quality of life) are needed.

The study findings demonstrate the significant strengths of specialty pediatric hospitals in terms of clinical attitudes and practice standardization for GAS pharyngitis diagnosis and treatment. This advantage likely stems from specialized pediatricians’ more frequent exposure to relevant cases and receipt of more systematic and focused professional training (Casimir, 2019). Thus, multidisciplinary team deployment in general hospitals is recommended.

This study has several limitations. Firstly, the inclusion of only a subset of clinicians in Shenzhen, coupled with the relatively small sample, restricts the generalizability of the results to broader clinical populations across China. Moreover, Shenzhen’s primary healthcare system (e.g., community hospitals) possesses diagnostic and management capabilities superior to those of many other urban centers in China (Li et al., 2020), further constraining the broader national applicability of the findings. Secondly, the voluntary nature of participation and distribution of questionnaires via experts’ networks potentially introduced selection bias. Specifically, the participating clinicians may have had a higher level of attention to guidelines, which could have led to the overestimation of knowledge levels. Thirdly, the KAP findings were derived entirely from self-reports, and no objective verification was performed. This approach is inherently susceptible to information bias arising from participants’ cognitive or social desirability biases. Finally, the univariate and multivariate regression analyses did not include all study variables. Key potential confounding factors, such as specific environmental influences and patient-related factors, were not accounted for in the models, likely leaving residual confounding that may have influenced the observed associations. To comprehensively characterize regional variations in clinicians’ knowledge and practices regarding GAS pharyngitis across China, multicenter studies conducted with geographically diverse cohorts and the representation of understudied regions (e.g., western region of China) are needed.

## 5 Conclusion

This study showed that clinicians in Shenzhen possess sound knowledge about and positive attitudes toward the diagnosis and management of GAS pharyngitis in children. However, although their level of knowledge appears to be influenced significantly by their exposure to systematic diagnostic and treatment guidelines, their clinical behaviors are demonstrably constrained by reliance on individual experience. Additionally, practices such as empirical therapy, routine PST and inadequate awareness of high macrolide resistance rates in China may exacerbate antibiotic resistance. To enhance guideline adherence and promote high-quality, evidence-based healthcare delivery, the strengthening of the standardized application of established diagnostic and therapeutic guidelines and the concurrent improvement of clinicians’ practical competencies through targeted educational interventions are recommended.

## Data Availability

The original contributions presented in the study are included in the article/supplementary material. Further inquiries can be directed to the corresponding author/s.
