# Correction: Knowledge, attitudes and practices of clinicians in Shenzhen regarding pediatric streptococcal pharyngitis diagnosis and treatment

**DOI:** 10.3389/fcimb.2026.1822360

**Published:** 2026-05-05

**Authors:** Shuting Zhuang, Danchun Guo, Yifan Ruan, Li Li, Yanmin Bao, Wenjian Wang, Dingle Yu

**Affiliations:** Shenzhen Children’s Hospital, Shantou University Medical College, Shenzhen, China

**Keywords:** child, group A *streptococcus*, knowledge, attitudes and practices, pharyngitis, Shenzhen

There are errors in **Table 5** “Analysis of Factors Influencing the Behavioral Dimension”:

The sample size for “Class I” in the “Hospital Grade” grouping has been corrected from “151” to “38”;

The sample size for “Class III grade-A” has been corrected from “38” to “151”.

There are errors in **Table 9** (Correlation Between GAS Pharyngitis Recurrence Frequency and Tonsillectomy):

Correct the number of participants in the “1-3” group who were “Tonsillectomy non-recommendations (n)” from “146” to “73”;

Correct the number of patients in the “4-6” group for whom “Tonsillectomy non-recommendations (n)” from “38” to “19”;

Correct the number of patients in the “7-10” group for whom “Tonsillectomy non-recommendations (n)” from “8” to “4”;

Correct the number of individuals in the “> 10” group for whom “Tonsillectomy non-recommendations (n)” from “14” to “7”;

Correct the total number of individuals for whom “Tonsillectomy non-recommendations (n)” from “206” to “103”.

The following sections have been updated:

There was a mistake in **Table 6** as published. “0.048*”. The corrected **Table 6** appears below.

“0.048”

There was a mistake in **Table 6** as published. “**Two-tailed *p* < 0.01, *one-tailed *p* < 0.05.”. The corrected **Table 6** appears below.

“**Two-tailed *p* < 0.01.”

In the *Abstract*, “Knowledge scores correlated with attitude and practice scores (*p* < 0.05), but attitude and practice scores did not correlate *(p* > 0.05)”. This has been corrected to read:

“Knowledge score correlated with practice score (r = 0.351, *p* < 0.05), but attitude score did not correlate with knowledge score (r = 0.048, *p* > 0.05) and practice score (r = 0.072, *p* > 0.05)”

A correction has been made to the section [Name of Section, subsection Name of Sub-section if there is one, Paragraph Number]:

Significant positive correlations of the knowledge score with the attitude (*p* = 0.048) and practice (*p* = 0.0035) scores were identified. No significant correlation was found between the attitude and practice scores (*p* = 0.072; **Table 6**).

“A significant positive correlation between the knowledge and practice scores was identified (r = 0.351, *p* < 0.05). No significant correlation was found between the attitude and knowledge scores (r = 0.048, *p* > 0.05), nor between the attitude and practice scores (r = 0.072, *p* > 0.05; **Table 6**)”

The original version of this article has been updated.

**Table 5:** Factors associated with practice scores.

Predictor	n	Score (mean ± SD)	Univariate analysis results		Multivariable linear regression results		
			F/t	p	b	95% Cl	p
Sex			-0.66	0.739			
Male	66	6.41 ± 0.88					
Female	124	6.49 ± 0.86					
Age group, years			1.09	0.338			
0-29	20	6.55 ± 0.69					
30-50	152	6.42 ± 0.90					
51-90	18	6.72 ± 0.75					
Highest degree			0.49	0.611			
Bachelor’s	77	6.39 ± 0.79					
Master’s	102	6.52 ± 0.92					
Doctorate	11	6.45 ± 0.82					
Years of work experience			2.74	0.030			
1-3	26	6.69 ± 0.74			-0.02	-0.46, 0.37	0.829
4-5	15	6.20 ± 1.01			-0.16	-1.02, -0.03	0.038
6-10	42	6.33 ± 0.98			-0.18	-0.74, -0.01	0.042
11-20	64	6.33 ± 0.82			-0.21	-0.70, -0.05	0.025
> 20	43	6.74 ± 0.76			0.000		
Hospital classification			3.21	0.043			
Class I	38	6.21 ± 0.70			-0.13	-0.59, 0.03	0.073
Class II	1				-0.12	-3.14, 0.19	0.081
Class III grade-A	151	6.54 ± 0.89			0		
Exposure to relevant guidelines/expert consensus			4.17	0.043			
No	62	6.27 ± 0.89			0		
Yes	128	6.55 ± 0.84			0.11	-0.06, 0.47	0.120

**Table 6:** KAP score correlations.

	Knowledge score	Attitude score	Practice score
Knowledge score	1.00	0.048	0.351**
Attitude score	0.048	1.00	0.072
Practice score	0.351**	0.072	1.00

**Two-tailed p < 0.01.

**Table 9:** Association between pediatric GAS pharyngitis recurrence frequency and tonsillectomy.

Annual pediatric GAS pharyngitis recurrence episodes (n)	Tonsillectomy recommendations (n)	Tonsillectomy non-recommendations (n)
1–3	18	73
4–6	7	19
7–10	2	4
> 10	2	7
Total	29	103

